# Effects of race distance and probiotics intervention on kidney, muscle, and gut injury and inflammation biomarker responses during running

**DOI:** 10.1080/15502783.2026.2651356

**Published:** 2026-04-14

**Authors:** Erik Hansson, Bethany Skinner, Tiziana Falcone, Amitava Halder, Rebekah A.I. Lucas

**Affiliations:** a Occupational and Environmental Medicine, School of Public Health and Community Medicine, University of Gothenburg, Gothenburg, Sweden; b La Isla Network, Washington, DC, USA; c School of Sport, Exercise and Rehabilitation Sciences, University of Birmingham, Birmingham, United Kingdom; d Unit of Advanced Robotics and Human-Centred Technologies, Campus Bio-Medico University of Rome, Rome, Italy; e Italian Implantable Prostheses Registry, Italian National Institute of Health, Rome, Italy; f Department of Experimental Medical Science, Faculty of Medicine, Lund University, Lund, Sweden; g Occupational and Environmental Medicine, Region Skåne, Lund, Sweden; h Department of Experimental Design Sciences, Faculty of Engineering, Lund University, Lund, Sweden

**Keywords:** Kidney, probiotics, exercise-induced organ stress, intestinal permeability, gut–kidney axis

## Abstract

**Introduction:**

Prolonged intense physical activity, such as long-distance running, may lead to systemic inflammation and cause organ injury, particularly to the kidneys. This study aimed to assess the impact of trail race running on kidney and muscle injury and gut inflammation biomarkers, and the potential mitigating effects of *Lactiplantibacillus plantarum* 299v (Lp299v) supplementation.

**Methods:**

This randomized, double-blind, placebo-controlled study included 34 participants who completed 42 races, ranging from 20 to 164 km. Participants were divided into two groups, receiving either 40 bn CFU Lp299v or a placebo for 4 weeks before the race. Urine and fecal samples were collected pre- and post-race (immediately after, morning after and 24 h after the race) to measure biomarkers of muscle and kidney injury, and gut inflammation. Principal component analysis was used to create a single tubular kidney injury biomarker component variable (TKIBC1) positively associated with five separate tubular kidney injury biomarkers (MCP-1, KIM-1, GST-*π*, clusterin, and calbindin) at the three post-race time points.

**Results:**

Running led to increased tubular and glomerular kidney injury markers, increased levels of fecal calprotectin, and, in some cases, elevated urine myoglobin levels. These effects were more pronounced in races ≥107 km (ultradistance). While Lp299v supplementation did not significantly influence TKIBC1, it was associated with a protective effect against gut inflammation.

**Conclusion:**

These findings suggest that prolonged intense exercise induces kidney and muscle injury as well as gut inflammation, with more severe effects observed in ultra-distance running. Lp299v may have some protective effects, particularly against gut inflammation, but further studies are needed to confirm these findings and explore the underlying mechanisms linking gut health and kidney injury during extreme physical exertion.

## 1. Introduction

There is a growing interest and participation in long-distance trials, with the number of participants doubling in the past 10 years [1]. However, the potential health consequences are poorly understood. Intense, prolonged physical activity, such as long-distance running, often leads to elevated serum concentrations of systemic inflammation biomarkers [2]. Similarly, markers of injury to organs such as the kidneys often increase [2–7]. The pathophysiology underlying this systemic inflammation and organ injury may partly precede or overlap with heat stroke: increased intestinal permeability leads to endotoxemia and systemic inflammation with organ injury [8,9]. The interplay between intestinal and other organs (e.g. kidney) inflammation and injury during prolonged intense physical activity is not well understood. However, this interplay may be relevant for both an increasing number of recreational ultra-distance runners and heat-stressed workers performing in a warming world.

Moreover, how the intestinal barrier function is affected by the gut microbiota is incompletely understood. Several studies have previously shown that probiotic bacteria may positively impact the gut barrier and decrease inflammatory levels in both healthy and diseased individuals [10–12]. However, the connection between exercise-induced gut permeability and inflammation is a new area of research. The International Society of Sports Nutrition considers that “some probiotic strains can improve the integrity of the gut-barrier function in athletes”, but that a large heterogeneity in probiotic strains used in studies means that firm conclusions are difficult to reach [13]*. Lactiplantibacillus plantarum* 299v (Lp299v) is a well-known probiotics strain, which has been shown to improve iron absorption in female professional athletes [14] and reduce intestinal permeability in septicemic rats [15]. Multi-strain probiotic supplementation (containing *Lactiplantibacillus plantarum*) tended (albeit not to reach statistical significance) to reduce lower gastrointestinal permeability and serum endotoxin concentrations before and after a short-term heat treatment [16]. In another study*, Lactiplantibacillus plantarum* PS128 supplementation decreased urinary myeloperoxidase and complement 5 levels while increasing the levels of the antioxidant thioredoxin in urine in response to a triathlon [17].

The aims of the present study were to assess 1. whether recreational race runners had an increase in biomarkers of tubular and glomerular kidney as well as muscle injury and gut inflammation, 2. if such biomarker responses were higher in ultradistance (107–164 km) compared to long-distance (20–55 km) races, and 3. if Lp299v supplementation 4 weeks before the race ameliorated biomarker responses.

## 2. Methods

### 2.1. Design of the study

The study was randomized, double-blinded, and placebo-controlled (ClinicalTrials.gov ID: NCT04985292), approved by the Swedish Ethical Review Authority (2021-01332) and conducted in Sweden. All participants provided signed informed consent before randomization into one of the two study groups.

Runners were recruited from May 2022 to September 2022 at races, training sessions and via social media forums. The included runners had to have run at least 21 km previously, not smoke and agree to abstain from probiotic supplements, including kimchi and functional foods containing probiotics in the 6 weeks preceding a race. Twenty-six of the recruited runners were already planning and training for gravel/trail races in southern Sweden (low-land and moderately hilly) during 2022. Six recruited runners ran in two races organized by researchers for the purpose of the study.

All statistical analyses were performed using Stata version 18 (StataCorp LLC, Texas, USA).

### 2.2. Sample and data collection

The participants were requested to provide fecal samples before the initiation of probiotics or placebo intervention, the day before or the same day of the race, and the day after the race. Fecal samples were collected by the participant in a plastic container, frozen in the participants’ home freezer (typically −20 °C), and picked up by the researcher as soon as logistically feasible (typically within 1–3 days) for transfer via a −20 °C freezer to a −80 °C freezer within days or a few weeks. No preservation media were used. Fecal calprotectin has been shown to be stable under these conditions over similar durations [18]. The participants provided urine samples immediately before and after the race. These samples were aliquoted within 10 min of voiding and placed on dry ice. Depending on logistics, samples can be kept in a dry ice container for up to 48 h before being placed in a freezer. The participants were requested to aliquot and put their urine samples from the morning after the race and 24 h after the race finished in their home freezer. They were requested to do so immediately after voiding. Urine samples were collected and maintained frozen by transport on dry ice and via a −20 °C freezer transferred to a −80 °C freezer within days or a few weeks.

Runners were asked to fill out a visual analogue scale to grade their overall gastrointestinal symptoms during the four-week pre-treatment period and race running, in comparison to their normal experience.

Urine samples were also collected from participating runners who had finished their probiotics/placebo intervention and were participating in later races the same season (“Race after trial” column in Table 2). These opportunistic urine samples were not used for analyzing probiotic effects but for exploring associations between race distance and kidney outcomes.

### 2.3. Intervention/exposure variables

#### 2.3.1. Intervention

The participants were randomly assigned to one of two arms: Lp299v or placebo (1:1) by the order in which they were recruited. The randomization list was computer-generated with a block size of four by an external statistician. The probiotic arm received one capsule daily, consisting of Lp299v potato starch, maltodextrin (bulking agents), and magnesium stearate (processing and anti-caking aid). The study products were manufactured specifically for the study by Probi USA, Lafayette, CO, USA. At the time of manufacture, the potency was confirmed by the manufacturer to be 40 billion colony-forming units (CFU). The study products were stored at 5 °C by the company to ensure stability. Tests after study completion verified that the stored capsules contained >10 billion CFU. Study products were typically handed out to runners a few weeks before their treatment period began, and they were instructed to keep the capsules in their refrigerator. The placebo arm received one capsule per day of identical appearance containing only potato starch and magnesium stearate. Both capsules were white vegetable capsules (hydroxypropyl methylcellulose and titanium dioxide). The study product was packed externally, and external personnel not involved in the study were responsible for labeling. The participants or study personnel involved did not know which product (Lp299v or placebo) was distributed. Runners were requested to abstain from non-steroidal anti-inflammatory drugs (NSAIDs) the 4 days preceding the race.

#### 2.3.2. Race characteristics

**Figure 1.**
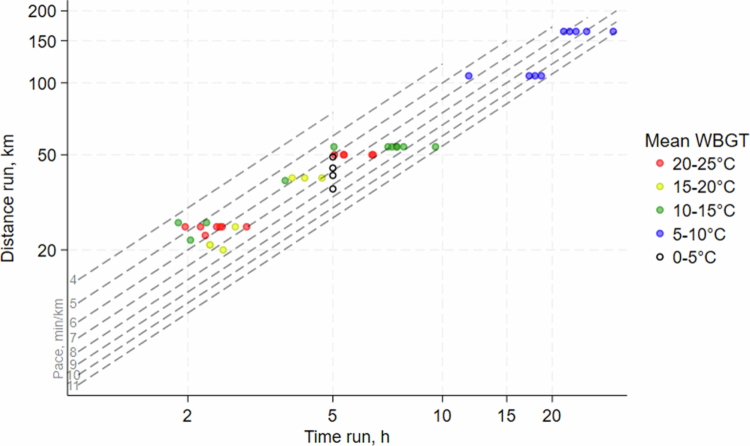
Distances, time, pace, and wet-bulb globe temperatures (WBGT) at off-road, trail run races (each dot is one runner-race).

Wet-bulb globe temperature (WBGT) was measured using a heat stress monitor (HSM 100, Casella Cel Limited, Bedford, UK) both in the sun and in the shade during the race, with a time weighted average calculated according to the proportion of cloud and vegetation cover (as assessed by the researcher). The distance of each of the fixed-distance runs was obtained from the race organizer. For races conducted over a fixed time rather than distance, the distance recorded by the runner’s GPS watch was used. Race distances were categorized into 20–25, 39–54, and 107–164 km, forming three distinct group of distances (Figure 1).

### 2.4. Outcome variables

#### 2.4.1. Urine concentration

Urine creatinine was measured at the Sahlgrenska University Hospital Clinical Chemistry Laboratory using an Alinity c instrument (Abbott Laboratories, USA) as a marker of urine concentration.

#### 2.4.2. Tubular kidney injury

Six tubular injury markers, namely, clusterin, calbindin, interleukin 18 (IL-18), glutathione S-transferase *π* (GST-*π*), kidney injury molecule 1 (KIM-1), and monocyte chemoattractant protein 1 (MCP-1) were measured in urine using BioRad’s Kidney Toxicity Panel I. Analyses were performed according to the manufacturer’s instructions. Four separate plates were used to run the samples. To reduce batch effects, we predominantly analyzed the four different samples collected from the same runner at each specific race using the same plate. IL-18 was below the limit of detection (LOD) for more than 50% of the time and was excluded from further analysis.

#### 2.4.3. Glomerular kidney injury

Urine albumin was measured at the Sahlgrenska University Hospital Clinical Chemistry Laboratory using an Alinity c instrument.

#### 2.4.4. Muscle injury

Urine myoglobin concentrations in the morning following the races were measured at the Sahlgrenska University Hospital Clinical Chemistry Laboratory using a CobasPro e801 instrument (Roche Diagnostics, Switzerland).

#### 2.4.5. Gut inflammation

Fecal calprotectin was measured using a Phadia250 instrument (Thermo Scientific).

### 2.5. Statistical analysis

#### 2.5.1. Tubular kidney injury

To reduce the number of outcome comparisons while simultaneously utilizing data on all tubular injury biomarkers, we decided on the following approach for combining the biomarkers into one composite outcome representing the overall tubular injury biomarker response:

First, mixed effects tobit regression models were run, modeling the log-transformed urine concentration of each biomarker while adjusting for log urine creatinine, analyzing the batch and timing of sampling in relation to race. Tobit regression was used to account for values below the LOD. The models had a random intercept for runner-race and a random slope for the pre- and post-race concentration changes. The random slope was estimated for each individual and biomarker, providing an estimate of the change in the biomarker across the race within that individual during that race.

Expectedly, the random slopes for all biomarkers were correlated (Supplement Table 1). A principal component analysis was then performed to generate a single variable for each runner-race, representing the magnitude of the increase in tubular kidney injury biomarkers during the race. Henceforth, this variable is denoted as Tubular Kidney Injury Biomarker Component 1 (TKIBC1). TKIBC1 was expectedly positively correlated with all the biomarkers’ random slopes, with correlation coefficients ranging between 0.77 and 0.83 with each of the individual biomarker random slopes (Supplement Table 1). This variable was also positively associated with creatinine-adjusted pre- and post-race comparisons for all single tubular injury biomarkers at every time point after the race (Supplement Figure 1). Comparisons of TKIBC1 across intervention groups and race characteristics were performed using graphical visualization and non-parametrical equality-of-medians tests.

TKIBC1 was calculated and compared across three different types of race groupings:

1. All races, including those run months after the placebo/probiotics intervention had finished. *N* = 40 races in 32 individuals.
2. 20–164 km races are immediately preceded by placebo/probiotics intervention. *N* = 32 races in 32 individuals.
3. 20–54 km races are immediately preceded by placebo/probiotics intervention. *N* = 26 races in 26 individuals.

The association between race distance and TKIBC1 were investigated in group 1, and the effect of probiotics were investigated in groups 2 and 3. The separation into groups 2 and 3 was performed as a few runners ran ultradistances in their first race. These more often received probiotics rather than placebo (Table 1) and were considered at a higher kidney injury risk.

**Table 1.** Demographic and running race characteristics.

		Race in trial^1^		Race after trial
		Placebo	Probiotics	Repeated race by trial participant
Number of participants		14	18	8
Age (years)		44 (35–48)	45 (35–49)	46 (39–51)
% of female (%)		43%	22%	13%
Distance (km)		33 (25–54)	43 (25–54)	50 (38–81)
% of distance (%)	20–25 km	50%	28%	25%
	36–55 km	36%	50%	50%
	107–164 km	14%	22%	25%
Time (hours)		3.2 (2.3–7.5)	5 (2.9–9.6)	5.2 (3.5–12.3)
Pace (km/h)		10 (8–11)	9 (7–9)	10 (7–10)
WBGT (°C)		16 (11–21)	13 (9–21)	12 (9–18)

Age, distance, time, pace, and WBGT were reported as median (IQR).

^1^ Two participants were excluded from the urine-based analyses because of specimen mishandling. “Race in trial” denotes endurance competitions that participants performed immediately after probiotics/placebo treatment. “Race after trial” denotes endurance competitions performed months after finishing probiotics/placebo treatment.

The effect of probiotic treatment and distance on each tubular injury biomarker were also assessed separately to test the intervention effect and to describe the association with race distance. This was accomplished by including an interaction effect for post-race sampling and probiotics intervention status and distance category in each of the above regression models. Random slopes were excluded from these models.

#### 2.5.2. Glomerular kidney injury

Log-transformed urine albumin concentrations were modeled using mixed effects linear regression, including fixed effects for the timing of sampling and log-transformed urine creatinine concentrations. Every runner-race had a unique random intercept. Interaction effects between post-race sampling and probiotic intervention and race distance were included in separate models to test the intervention effect and to describe the association with race distance.

#### 2.5.3. Gut inflammation

Log-transformed fecal calprotectin concentrations were modeled using mixed-effects tobit regression, including fixed effects for the timing of sampling. Every runner-race had a unique random intercept. Interaction effects for post-race sampling, probiotics intervention and race distance were included in separate models to test the intervention effect and to describe the association with race distances.

## 3. Results

Out of 42 recruited runners, 34 runners started and finished a total of 42 races. Six participants did not start the race, and two others started but did not finish their races. Two out of the 34 participants did not follow the instructions on freezing urine samples immediately after voiding on the day after the race, and these participants were excluded from all the urine-based analyses. Four participants refused fecal sampling, and of the remaining thirty participants, five participants did not provide pre-intervention fecal samples for logistical reasons. All races were predominantly conducted on gravel roads, tracks, or trails and over very limited distances on asphalt. The race distances ranged between 20 and 164 km, with running times ranging between 2 and 30 h. Most participants were male (69%) and the median age was 44 years (Table 1). The environmental conditions during the race varied substantially, ranging from 20 to 25 °C WBGT on a sunny day in August to approximately 0 °C WBGT in early November (Figure 1). Race distances and pace also differ substantially, ranging from 20 to 164 km and from 4 to almost 11 min/km (Figure 1).

### 3.1. Probiotic effect

**Figure 2.**
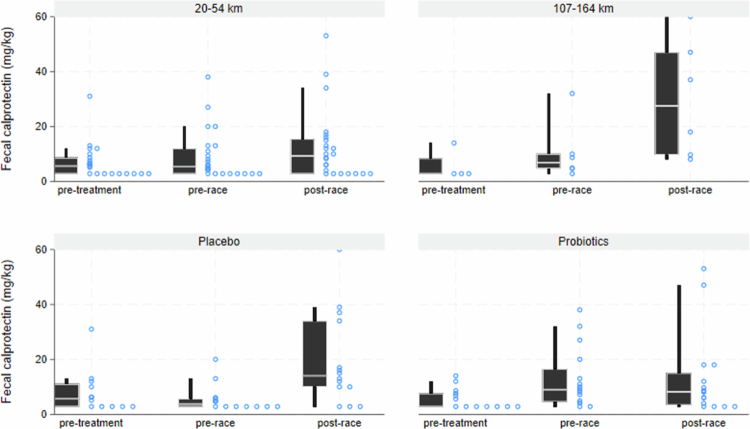
Fecal calprotectin levels by time of sampling, trail race distance, and probiotics intervention status. Boxes describe median, 25^th^ and 75^th^ percentiles (interquartile range), and whiskers represent the 10^th^ and 90^th^ percentiles. Individual calprotectin levels are shown as circles.

**Figure 3.**
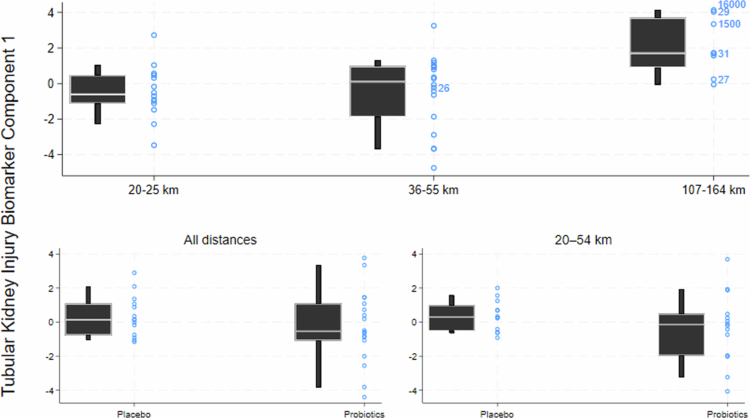
Tubular kidney injury biomarkers component 1 (TKIBC1, a composite index representing the magnitude of tubular kidney injury biomarker rise during the race) by race distance and probiotics intervention status. Boxplots are showing median, 25^th^ and 75^th^ percentiles (interquartile range), and whiskers represent the 10^th^ and 90^th^ percentiles. Individual TKIBC1 levels are shown as circles. The values to the right of the circles indicate measurable urine myoglobin levels (µg/L). Circles with no values have myoglobin levels below the limit of detection (<25 µg/L).

Runners randomized to take a probiotics supplement had nominally smaller increases in tubular injury markers during the race. However, no statistically significant effect was found for any of the individual markers (smallest *p* = 0.07, for MCP-1 in 20–54 km races only (Table 2)). The increase in the combined tubular injury biomarker variable, TKIBC1, was nominally lower in runners taking probiotics in the four weeks preceding the race compared to placebo (bottom panels in Figure 3). However, the differences did not reach statistical significance (*p* = 0.48 for all races, *p* = 0.12 if including only 20–54 km races). Probiotics protected against elevated fecal calprotectin compared to pre-race values (*p* < 0.001) (Figure 2). However, probiotics also led to an increase in fecal calprotectin during the intervention period (*p* = 0.01) (Figure 2).

**Table 2.** Mixed effects tobit regression coefficients (with 95% confidence intervals [C.I]) for kidney injury biomarkers at different sampling time points.

Kidney injury type	Marker	Pre-race	Immediately post-race, *β* (95% C.I.)	Morning after race, *β* (95% C.I.)	24 h after race finishes, *β* (95% C.I.)
Tubular	Clusterin	Reference	0.96 (0.52–1.40)	0.31 (−0.16–0.77)	0.30 (−0.14–0.74)
	Calbindin	Reference	0.93 (0.52–1.34)	−0.06 (−0.49–0.36)	0.02 (−0.38–0.42)
	GST-π	Reference	0.44 (−0.04–0.92)	0.31 (−0.20–0.83)	0.12 (−0.36–0.60)
	KIM-1	Reference	0.38 (0.03–0.74)	0.36 (−0.02–0.73)	0.19 (−0.17–0.54)
	MCP-1	Reference	0.58 (0.25–0.90)	0.33 (−0.02–0.68)	0.32 (−0.01–0.64)
Glomerular	Albumin	Reference	1.44 (1.13–1.76)	−0.01 (−0.33–0.31)	0.16 (−0.13–0.46)

Coefficients are for natural log-transformed biomarkers, adjusting for sample creatinine concentration and batch effects. A random intercept is included for each runner-race.

**Figure 4.**
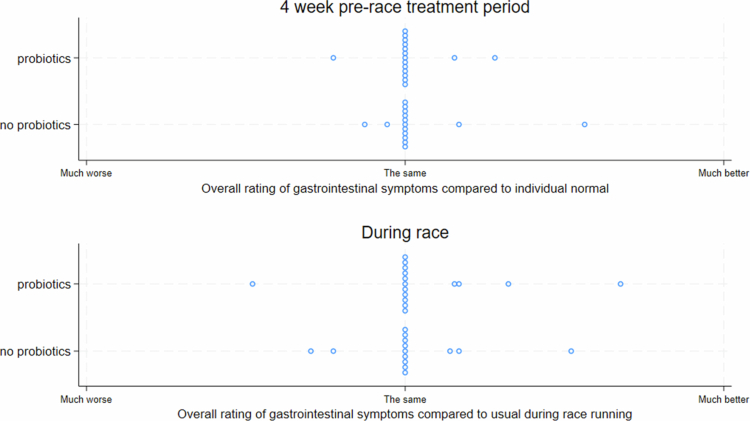
Summary of runners’ visual analogue scale reports for gastrointestinal symptoms during the treatment period and race running.

There was no difference in perceived symptoms during the pre-treatment period or in the race for runners receiving or not receiving probiotics (Figure 4).

### 3.2. Trail race effects

Race running led to an increase in all kidney injury biomarkers, which generally peaked immediately after the race (Table 2). Some markers, such as calbindin and albumin on average were normalized on average by the following morning, while MCP-1 and KIM-1 were slower to normalize. Fecal calprotectin was higher the day after than before the race (Figure 2, *p* = 0.001).

### 3.3. Trail race distance

Runners running ultra-distance races had larger increases in tubular and glomerular kidney injury markers than runners running shorter races (Table 3, Figure 3). In the morning after a ultra-distance race, 2 out of 9 runners had substantially elevated urine myoglobin (≥1500 µg/L), with another three runners having detectable but lower levels (26–31 µg/L), and the remaining four had < LOD (<25 µg/L). Runners with high myoglobin levels also presented markedly elevated tubular injury markers after the race (Figure 3). Urine myoglobin was only detectable in one runner after a 36–54  km race. Ultra-distance runners had a larger increase in fecal calprotectin levels after the race than runners running shorter distances (Figure 2, *p* = 0.03).

**Table 3.** Mixed effects tobit regression coefficients (with 95% confidence intervals) for kidney injury biomarkers describing effects of race distance and probiotics intervention on pre/post-race effects.

		Races preceded by probiotics/placebo intervention			
		20–164 km	20–54 km	All races	
Kidney injury type	Marker	Intervention effect^2^	Intervention effect^2^	Main pre/post-race effect	Interaction effect for ultra-distance^1^ category
Tubular	Clusterin	−0.31 (−1.12–0.49)	0.03 (−0.89–0.96)	0.53 (0.15–0.92)	1.16 (0.26–2.07)
	Calbindin	−0.60 (−1.33–0.13)	−0.53 (−1.39–0.33)	0.32 (−0.04–0.66)	0.68 (0.01–1.34)
	GST-π	−0.53 (−1.32–0.25)	−0.40 (−1.31–0.51)	0.28 (−0.11–0.68)	1.03 (0.21–1.84)
	KIM-1	−0.25 (−0.82–0.32)	−0.15 (−0.82–0.52)	0.30 (0.00–0.59)	0.78 (0.11–1.46)
	MCP-1	−0.31 (−0.86–0.25)	−0.57 (−1.17–0.04)	0.41 (0.14–0.68)	0.94 (0.32–1.57)
Glomerular	Albumin	0.15 (−0.52–0.82)	0.31 (−0.41–1.02)	0.43 (0.10–0.77)	1.12 (0.40–1.85)

Coefficients are for natural log-transformed biomarkers, adjusting for sample creatinine concentration and batch effects. A random intercept is included for each runner-race.

^1^ 107–164 km vs. 20–54 km race distance.

^2^ Interaction coefficient for probiotic intervention at post-race sampling.

## 4. Discussion

### 4.1. Key results

Trail race running for longer than 20 km induced an increase in biomarkers of kidney injury (clusterin, calbindin, KIM-1, and MCP-1) as well as gut inflammation (calprotectin) in most participating runners. Running ultradistances were associated with a greater increase in markers of kidney and muscle injury as well as gut inflammation. The effect of probiotics was protective against calprotectin and tubular injury markers, but did not reach statistical significance for the latter.

### 4.2. Strengths and limitations

The current study successfully recruited and tested 32 runners who underwent a 4-week nutritional intervention. Furthermore, over 80% of the participants ran in officially organized races (i.e. not simulated), enhancing the external validity of our findings. A strength of collecting samples from real races is that participants are conceivably more motivated/willing to exercise for longer/harder intensities. This is important given the likely role exercise/work intensity and duration have on kidney and gut health.

Recruitment challenges determined that runners had to be recruited from different races over a period with varying climatic conditions. Thus, a heterogeneous range of races was unavoidable. The length of the races provided an opportunity to describe the impact of race distance on kidney and gut outcomes. However, homogenous races (i.e. distance, weather conditions) likely would have increased the power to discern a probiotics intervention effect by reducing some of the variability in the present study.

The results were adjusted for urine creatinine, as it is customary when adjusting for concentrations in spot samples [19]. However, using creatinine adjustment introduces a risk for bias toward underestimating the effect of ultra-distance running, as creatinine synthesis and turnover are expected to be greater over longer distances [6].

Future studies seeking to prevent kidney injury among race runners could learn from this study. Firstly, to recruit enough runners, sample collection needs to be simple and acceptable. In our experience, fecal sampling deterred a substantial number of participants, whereas urine sampling was perceived as convenient and generally acceptable. Nevertheless, fecal samples represent a non-invasive way of assessing gut inflammation, which is an important outcome in these circumstances. Providing participants with options for what kind of samples they are willing to contribute could aid in recruitment. Secondly, the duration of the nutritional/supplement intervention is important for participant recruitment. The necessary duration of probiotics pre-treatment ahead of a race is unknown, and may also vary by strains. Future research with different treatment durations and strains could identify optimal therapeutic approaches. Measuring and/or standardizing important dietary aspects, e.g. fiber content, may also improve the ability to study probiotics effects.

### 4.3. Interpretation

Race running leads to a clear and statistically significant increase in several tubular injury biomarkers, similar to the findings of previous studies of marathon runners [3,7]. We also found that MCP-1 levels remained elevated the day after the race, while KIM-1 levels had almost normalized 24 h after the race. Previous studies examining kidney outcomes in marathon runners and ultra-marathon runners have primarily used serum creatinine or urine dipstick measures. These studies have generally detected increasing levels during races [2,5,20], although with some exceptions [21].

This study provides further evidence of an increase in kidney injury biomarkers after long-distance running, with a dose‒response relationship observed with race distance. Ultra-distance races were associated with a larger increase in kidney as well as gut markers of inflammation and injury than shorter long-distance races. This finding is congruent with a previous study that revealed a larger increase in circulating markers of systemic inflammation following ultra-marathon compared to marathon races [22].

To our knowledge, no previous studies have measured fecal calprotectin levels across half-to-ultra marathon distances. A previous study that examined the effects of 90 min running (approximately 17 km) on fecal calprotectin levels reported no increase [23]. This finding is compatible with our findings of a smaller increase in fecal calprotectin after short-distance (20–54 km) races.

Muscle injury was also more prevalent following longer-distance races. 2 out of 9 ultra-distance runners had markedly elevated urine myoglobin levels, and the majority had detectable urine myoglobin levels. This finding is in line with a previous study in 99 km race runners [24], indicating that a rhabdomyolysis-like mechanism of kidney injury occurs relatively frequently at such extreme distances.

Another possible cause of kidney inflammation and injury is reduced gut barrier function during prolonged and/or strenuous exercise, leading to endotoxemia and a systemic inflammatory response with associated organ inflammation and injury [25]. We were not able to sample blood or urine intermittently during the races and were thus not able to measure circulating endotoxin levels or gut barrier function in the present study. Instead, we relied on randomization of a probiotic supplement intended to strengthen the gut barrier and measurements of fecal calprotectin as a marker of gut inflammation for assessing this potential pathway.

Interestingly, Lp299v supplementation led to an increase in fecal calprotectin levels during the treatment but prevented a further increase during the race. This increase in calprotectin is contrary to the reductions observed in inflammatory bowel disease or cystic fibrosis patients randomized to probiotics supplementation [26,27], and the absence of effect in patients with irritable bowel disease dominated by diarrhea [28]. The effect of probiotics supplementation on fecal calprotectin levels in healthy, physically active individuals has not been previously studied. In a study on healthy older individuals with low-grade inflammation, the intake of another *L. plantarum* strain (HEAL9) led to a lower fecal calprotectin level [29]. The pattern observed here suggests that some young, healthy, and physically active individuals may experience somewhat increased calprotectin levels, at least during the initial phase of Lp299v treatment. A few runners reported an “upset stomach” after starting treatment, possibly indicating that they did not tolerate the supplement completely well. However, participants’ ratings of gastrointestinal symptoms did not differ between groups with and without probiotics pretreatment, with most runners describing no change in symptoms compared to usual runners (Figure 4). Differences in subjective and biomarker response to the supplement strain may have been modified by unmeasured factors such as diet (such as fiber content), pre-treatment gut microbiota or constitutional immunological factors. However, overall, when probiotics are used in a long- or ultra-distance race, those receiving probiotic supplements appear to be protected from increased gut inflammation. More studies are needed to replicate and potentially understand this phenomenon.

There is a tendency towards a protective effect of the probiotics supplement on the combined tubular kidney injury biomarker index. This protective effect differed between tubular injury markers, with the clearest effect observed for MCP-1, but it was in the protective direction for all five markers (except for clusterin in the 20–54 km group). This is compatible with compromised gut barrier integrity and resulting systemic inflammation as one possible mechanism behind kidney injury during physiological strain. The gut barrier integrity may be modified by the gut microbiota composition. Our results are in the same direction as those of the only previous study to our knowledge [17], showing a beneficial effect of *L. plantarum* supplementation ahead of intense physical activity on some kidney biomarkers. Beyond the scientific literature and reports of athletic use [13], it is common for manual workers in India to consume fermented buttermilk to alleviate heat stress [30]. This use of fermented buttermilk during work may stem from traditional knowledge on health benefits of lactic acid bacteria under these circumstances.

Understanding the gut‒kidney interplay during prolonged physical activity is important for athletes as well as working populations performing intense manual labor in the heat. Accumulating evidence has linked repeated heat stress-induced kidney injury with the epidemic of chronic kidney disease of non-traditional origin (CKDnt) [25,31]. Proteomic and metabolomic studies have identified pathways involving gut immunity, permeability and the microbiota as potentially disturbed in workers at risk of CKDnt [32–34]. An improved understanding of the interplay between the gut and kidney during intense exercise may contribute to important insights into CKDnt.

### 4.4. Conclusion

We found that race running induced an increase in biomarkers of kidney injury and gut inflammation. These biochemical responses were larger after ultra-distance races. Ultra-distance races also often induce myoglobinuria. Intervention with a *Lactiplantibacillus plantarum* 299v probiotic supplement for four weeks before a race decreased the gut inflammatory response during the race, and there was also a tendency towards a protective effect on tubular kidney injury biomarkers. This is compatible with an interplay between the gut and kidney during severe physiological strain, potentially mediated by increased gut permeability and resulting systemic inflammation. However, larger controlled studies are needed to understand this further.

## Supplementary Material

Supplementary material Supplement

## Data Availability

Data are available from the corresponding author upon reasonable request.
