# Supplementary material for: Effects of race distance and probiotics intervention on kidney, muscle, and gut injury and inflammation biomarker responses during running
Source: J Int Soc Sports Nutr. 2026 Apr 14;23(1):2651356. doi: 10.1080/15502783.2026.2651356 (PMC13084833; doi:10.1080/15502783.2026.2651356)

# Supplement

|  | CALBINDIN | CLUSTERIN | GST-π | MCP-1 | KIM-1 |
| --- | --- | --- | --- | --- | --- |
| CALBINDIN | 1.00 |  |  |  |  |
| Clusterin | 0.54 | 1.00 |  |  |  |
| GST-π | 0.49 | 0.71 | 1.00 |  |  |
| MCP | 0.55 | 0.30 | 0.55 | 1.00 |  |
| KIM-1 | 0.60 | 0.53 | 0.50 | 0.67 | 1.00 |
|  |  |  |  |  |  |
| Tubular Kidney Injury Biomarker Component 1 | 0.80 | 0.77 | 0.82 | 0.77 | 0.83 |

Supplement Table 1: Correlation between pre/post-race random slopes for 5 tubular injury biomarkers, and with their first component from a principal component analysis.


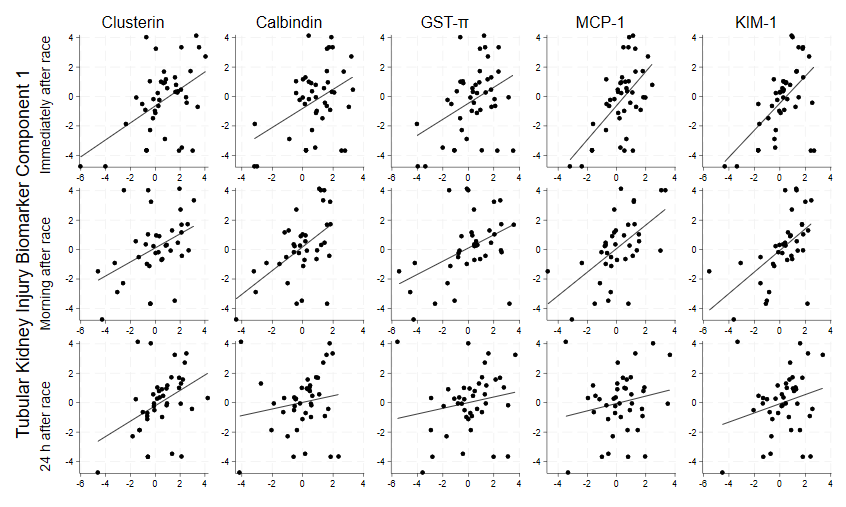

Supplement: Supplementary material — Supplement [file RSSN_A_2651356_SM0394.docx]
